# Nicolau syndrome following glatiramer acetate for multiple sclerosis: Case and review of reports

**DOI:** 10.1002/acn3.52044

**Published:** 2024-03-14

**Authors:** Methma Udawatta, Farrah J. Mateen

**Affiliations:** ^1^ Department of Neurology Brigham and Women's Hospital Boston Massachusetts USA; ^2^ Department of Neurology Massachusetts General Hospital Boston Massachusetts USA

## Abstract

Nicolau syndrome is a rare, iatrogenic skin reaction after parental drug administration, characterized by severe pain at an injection site, followed by hemorrhage, ulceration, and often necrosis. We present a case of a patient on glatiramer acetate for many years (initially Copaxone then Glatopa) who developed Nicolau syndrome, the second reported case after generic glatiramer acetate. All reported cases of Nicolau syndrome after glatiramer acetate are reviewed. The case highlights the importance of prompt recognition of this skin reaction by neurologists and raises awareness of the risks of skin reactions even in low‐risk injectable DMTs.

## Case Report

Our patient is a 37‐year‐old woman with relapsing remitting multiple sclerosis (RRMS), diagnosed in 2014. She was started on Copaxone (glatiramer acetate) thrice weekly subcutaneous injections in 2014, and she remained on this until 2019 (other than a combined total of 20 months discontinuation for two pregnancies). Her other past medical history is basal cell skin cancer (of her nose, resected), and her only other medication was a multivitamin. After 5 years on Copaxone, she transitioned to the generic version of glatiramer acetate, Glatopa. She had significant pain and bruising at injection sites throughout this time and developed skin indentations concerning for lipoatrophy even while rotating injection sites.

**Figure 1:**
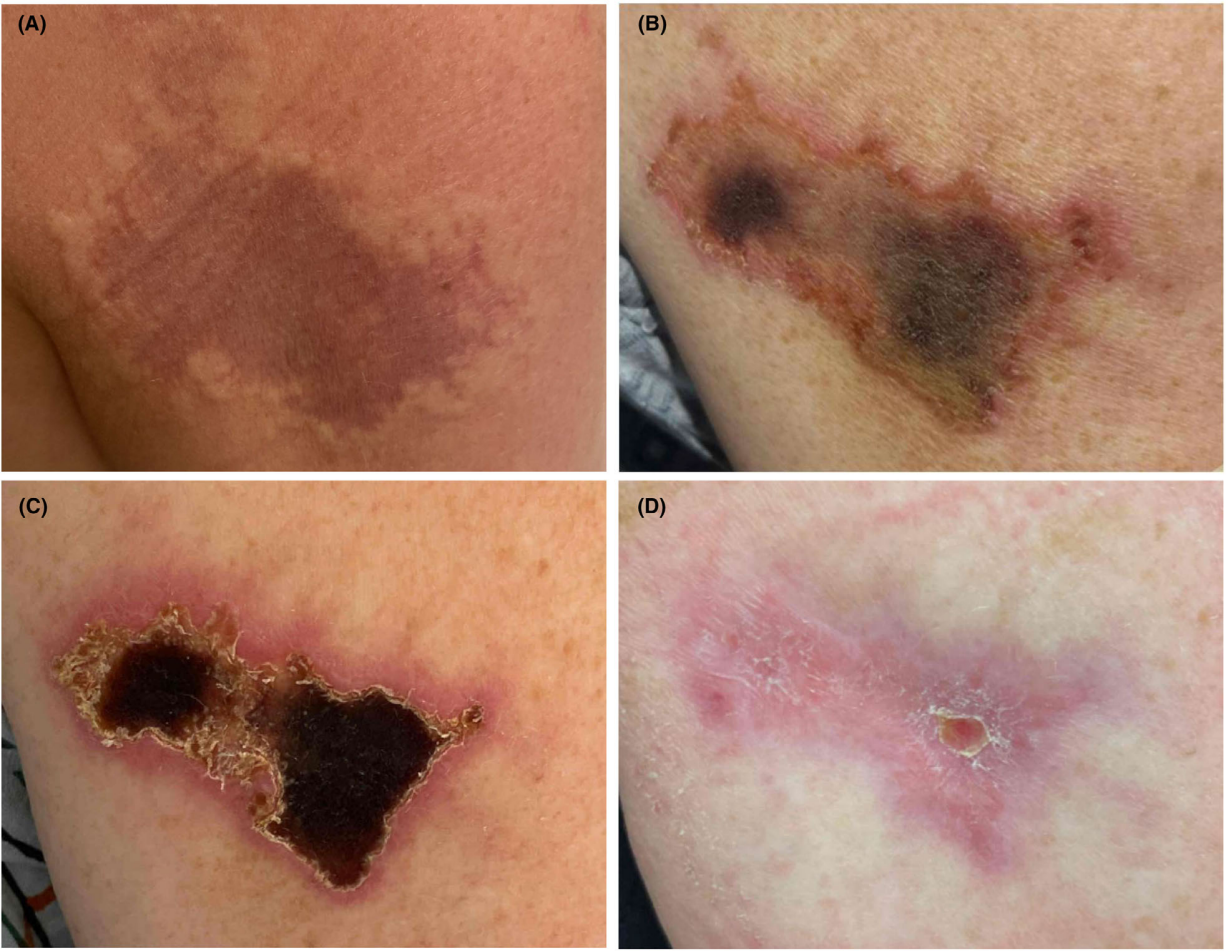
Images of Nicolau syndrome progression on initial discovery date (A), after 13 days (B), 18 days (C), and 89 days (D).

Four years into taking Glatopa regularly, the patient noticed new pain and swelling within 1 h of subcutaneous injection in the right arm. There were no concerns about injection technique. An image of her lesion is in Figure 1A.

Over the next 2 weeks, the lesion became more painful and necrotic (Fig. 1B,C) so she presented to the Emergency Department. Bedside ultrasound did not show any fluid pockets concerning for infection. She was seen by Dermatology 6–7 weeks afterwards who described a 5.7 × 4 cm lesion with pink scaling borders and hemorrhagic crusting at the center (1c), concerning for an injection site reaction consistent with Nicolau syndrome. Dermatology recommended wound care with mupirocin, pain control with ibuprofen, to avoid sprays or fragrances to the area until healed, and to follow‐up with a surgeon for potential wound debridement. Plastic surgery recommended conservative management, providing Santyl and wet‐to‐dry dressing changes for a month.

At a follow‐up visit 2 months from the start of her reaction, there was significant improvement in the size of the skin lesion. At this visit, it was described as “3.5cm × 2cm with healthy looking borders and no necrotic eschar.” The wound bed was debrided revealing “healthy pink granulation tissue.” Three months after the reaction, her wound was 1 cm × 1 cm with healthy looking borders and no necrotic eschar (Fig. 1D).

The patient discontinued Glatopa soon after developing her skin reaction and transitioned to Ocrevus (ocrelizumab). There was no new demyelinating disease activity manifested as a clinical relapse throughout the time she was on Copaxone, Glatopa, or Ocrevus. She experienced significant pain, disfigurement in the arm, stress, and additional out of pocket costs for health visits.

## Discussion

Nicolau syndrome (also called livedoid dermatitis or embolia cutis medicamentosa) is a rare iatrogenic dermatologic complication after parenteral drug administration. It classically occurs with intramuscular drug administration but is also seen with subcutaneous, intravenous, and intraarticular injection.^1^, ^2^ Nicolau syndrome was first described in 1924 and 1925 by Freudenthal and Nicolau, respectively, in the setting of intramuscular injection of bismuth salts.^3^, ^4^, ^5^ Reactions have been reported with a variety of drugs including antibiotics, NSAIDs, anesthetics, steroids, antipsychotics, and vaccinations.^6^

Glatiramer acetate has been approved for the treatment of RRMS by the U.S. FDA since 1996 with a generic equivalent approved in 2015. The medication has over 2 million patient‐years of exposure, reaching thousands of patients yearly. Approved for treatment of RRMS in 57 countries, glatiramer acetate was recently added to the World Health Organization's Essential Medicines List in 2023 and therefore may be prescribed with increasing frequency worldwide in the future.

Here, we present a case of Nicolau syndrome after generic glatiramer acetate (Glatopa) which to our knowledge is the second reported incidence of Nicolau syndrome after the generic glatiramer acetate. Similar to reported cases, our patient was treated with this DMT with usual injection techniques for several years (cases reported in the literature describe Nicolau syndrome after 10 months to greater than 10 years on injectable glatiramer acetate) (Table 1). Although our patient previously had developed lipoatrophy, there are no data on associations between Nicolau syndrome and previous lipoatrophy. Nicolau syndrome has been described as a reaction to glatiramer acetate and interferon‐beta but not to any other multiple sclerosis disease‐modifying therapies (DMTs).^7^ To date, there have been 34 reported cases of Nicolau syndrome associated with glatiramer acetate (Table 1). Most of these cases were in the setting of Copaxone use or were presumed branded drug, based on timing prior to generic glatiramer acetate approval.

**Table 1:** Patient demographics and characteristics of published cases of Nicolau syndrome after glatiramer acetate.

Study	Type of study	Number of patients	Age (years)	Sex (M, F)	Site of administration	Drug	Time on DMT prior to reaction	Glatiramer acetate continued?
Gaudez *et al*. (2003)^a^ ^,^ ^15^	Case report	1	33	F	Arm	Copaxone	10 months	Continued after brief interruption
Bosca *et al*. (2006)^a^ ^,^ ^16^	Case report	2	38	F	Abdomen	Copaxone	16 months	Switched to Azathioprine
			27	M	NR	Copaxone	18 months	Switched to IFN‐b1a
Harde *et al*. (2007)^a^ ^,^ ^17^	Case report	1	59	M	Abdomen	Copaxone	6 years	Continued
Feldmann *et al*. (2009)^a^ ^,^ ^18^	Case report	1	55	F	Abdomen	Copaxone	2 years	Continued
Koller *et al*. (2011)^a^ ^,^ ^19^	Case report	1	39	F	Abdomen	Copaxone	2 years	Continued
Martinez‐Moran *et al*. (2011)^a^ ^,^ ^20^	Case report	1	31	F	Buttock	Copaxone	4.5 years	Continued
Pulido Perez *et al*. (2013)^a^ ^,^ ^21^	Case report	1	45	F	Thigh	Copaxone	3 years	Continued
Samoes *et al*. (2014)^a^ ^,^ ^22^	Case series	4	35–43	F	Abdomen 3, Thigh 1	Copaxone	10 months–4 years	2 continued, 2 discontinued
Dorado Fernandez *et al*. (2015)^a^ ^,^ ^23^	Case report	1	48	M	Arm	Copaxone	4 years	Continued
Lobato‐Berezo *et al*. (2015)^a^ ^,^ ^24^	Case report	1	32	F	Abdomen	Copaxone	NR	NR
Zecca *et al*. (2015)^a^ ^,^ ^25^	Case report	1	58	F	Abdomen	Copaxone	9 years	Discontinued
Kimbrough *et al*. (2016)^a^ ^,^ ^26^	Case report	2	34, 43	F	Thigh	Copaxone	2 years, 3 years	Discontinued all DMT; Switched to Natalizumab
Mott *et al*. (2016)^a^ ^,^ ^27^	Case report	1	51	F	Abdomen	Copaxone	7 years	Discontinued
Blind *et al*. (2018)^28^	Case report	1	64	F	Suprapubic	Copaxone	7 years	Switched to Teriflunomide
Esme *et al*. (2021)^29^	Case report and review	1	34	F	Abdomen	NR	1.5 years	NR
Sy *et al*. (2021)^30^	Case report	2	62	M	Abdomen	Glatopa	5 years	Continued
			59	F	Hip	Copaxone	>10 years	NR
Vlahova *et al*. (2021)^a^ ^,^ ^31^	Case report	1	55	M	Abdomen	Copaxone	4 years	Discontinued
Ciprian *et al*. (2022)^32^	Systematic review	20	Mean 42 (range 34–42)	F:M 16:4	Abdomen 11, Thigh 5, Forearm 2, Buttock 1	NR		12/20 continued
Demircan *et al*. (2023)^a^ ^,^ ^33^	Case report	1	26	F	Thigh	NR	4 years	Continued
Neri *et al*. (2023)^34^	Case series	10	Mean 40 years	F:M 1:9	Abdomen 1, Arm 3, Buttock 4, Thigh 2	NR	Mean 6 years	NR

F, female; M, male; NR, not reported.

^a^ Included in Ciprian *et al*. systematic review.

Nicolau syndrome is characterized by severe pain at an injection site, followed by skin blanching, then an erythematous macule that becomes a livedoid violaceous patch over minutes to hours, followed by hemorrhage and ulceration at the lesion.^8^ This is often followed by necrosis, scarring, and possible infection. A diagnosis of Nicolau syndrome should be considered if patients develop a severely painful injection site reaction followed by skin changes including erythema, livedo reticularis, and hemorrhage at the site. There are case reports of peripheral neuropathies with sciatic neuropathies being a common source of injury after injection sites in the buttock; EMG/NCS showed severe axonopathy in one case.^9^ The differential diagnosis for a skin lesion after injectable DMT in MS should include the following (listed in descending order by number of patients affected): lipoatrophy, cutaneous ulcers, cutaneous necrosis, panniculitis, urticaria, cutaneous vasculitis, Nicolau syndrome, lupus‐like reactions, psoriasis, and allergic local reactions.^10^

The mechanism and pathogenesis of Nicolau syndrome is not well understood but some theories for its pathophysiology include embolic occlusion of arteries from injection, perivascular inflammation, mechanical injury to blood vessels, sympathetic nerve stimulation, or prostaglandin synthesis blockade (causing vasospasm and ischemia).^11^

There is no specific diagnostic test, so diagnosis is based on clinical presentation and exam. If skin is biopsied, histology often shows thrombosis of small‐ and medium‐sized vessels with inflammation.^12^

Treatment is generally supportive care, with concurrent use of intravenous anticoagulation, steroid, and vasodilators (although there are no definitive guidelines on these recommendations), and surgical consultation if there is concern for compartment syndrome or for debridement and skin grafts.^13^, ^14^ Recurrence occurs infrequently and in most reported cases of Nicolau syndrome after glatiramer acetate, patients continued the same medication (using different injection sites). However, a significant number of patients switched to a different DMT and one patient even discontinued DMTs altogether.^24^

## Conclusion

Nicolau syndrome is a rare but serious complication of subcutaneous glatiramer acetate, previously mainly described with brand name Copaxone, but now described for the second time in generic Glatopa, a commonly prescribed DMT for RRMS. It is important for neurologists to be able to recognize possible adverse reactions of DMTs, as patients will often contact their neurologist first for an injection site reaction. Prompt recognition of possible diagnoses can result in early dermatological and surgical evaluation for treatment options. Finally, it is important for neurologists to be aware of the possible side effects of medications otherwise thought to have a good safety profile, as these rare reactions can be disfiguring, can cause serious sequelae such as infection, and can lead to mistrust in the healthcare system and discontinuation of therapy.

Our patient gave her written informed consent to disclose her medical condition and related information along with the images used in the figure. CARE reporting guidelines were followed.

## Author Contributions

MU: Wrote first draft, data interpretation, editing for critical content, literature review. FJM: Study concept, data interpretation, editing for critical content, study supervision.

## Conflict of Interest

F. Mateen has received research funding from Genentech, Horizon Therapeutics, and Novartis and has consulted for Alexion, EMD Serono, Genentech, Horizon Therapeutics, and TG Therapeutics, all unrelated to this work. M. Udawatta received no specific grants from any funding agency in the public, commercial, or not‐for‐profit sectors.

## Data Availability

The data that support the findings of this study are available on request from the corresponding author, FM.
